# RepExplore: addressing technical replicate variance in proteomics and metabolomics data analysis

**DOI:** 10.1093/bioinformatics/btv127

**Published:** 2015-02-28

**Authors:** Enrico Glaab, Reinhard Schneider

**Affiliations:** Luxembourg Centre for Systems Biomedicine (LCSB), University of Luxembourg, Esch-sur-Alzette, Luxembourg

## Abstract

**Summary:** High-throughput omics datasets often contain technical replicates included to account for technical sources of noise in the measurement process. Although summarizing these replicate measurements by using robust averages may help to reduce the influence of noise on downstream data analysis, the information on the variance across the replicate measurements is lost in the averaging process and therefore typically disregarded in subsequent statistical analyses.

We introduce RepExplore, a web-service dedicated to exploit the information captured in the technical replicate variance to provide more reliable and informative differential expression and abundance statistics for omics datasets. The software builds on previously published statistical methods, which have been applied successfully to biomedical omics data but are difficult to use without prior experience in programming or scripting. RepExplore facilitates the analysis by providing a fully automated data processing and interactive ranking tables, whisker plot, heat map and principal component analysis visualizations to interpret omics data and derived statistics.

**Availability and implementation:** Freely available at http://www.repexplore.tk

**Contact:** enrico.glaab@uni.lu

**Supplementary information:** Supplementary data are available at *Bioinformatics* online.

## 1 Introduction

Technical noise is a common limitation in many high-throughput biological experiments. Both mass spectrometry devices for proteomics and metabolomics profiling as well as gene and protein microarray platforms can only provide a limited reproducibility (Albrethsen, 2007; Chen *et al.*, 2007). In combination with the biological variance observed across different omics samples under the same condition, the technical variability can significantly aggravate the statistical analysis of the data, increasing the risk for spurious and misinterpreted results.

A common approach to reduce the influence of noise on the statistical analysis of omics data is to use technical replicate measurements, e.g. for mass spectrometry data collecting three technical replicates per biological sample is a typical setting. During data pre-processing the replicate measurements are summarized to average values, by determining the mean, median or a trimmed mean, to reduce the influence of noise in downstream data analysis. However, the variance across replicate measurements often differs significantly between the biological samples and this data on measurement uncertainty is not retained by the summarization and consequently not considered in following statistical analyses.

Approaches to exploit technical variance information to improve robustness and sensitivity in downstream data analyses have been developed in recent years for differential expression analysis (Liu *et al.*, 2006), principal component analysis (PCA; Sanguinetti *et al.*, 2005) and differential pathway analysis (Glaab and Schneider, 2012). To enable users with limited or no programming experience to benefit from these new techniques to propagate variance information to downstream analyses, we have developed RepExplore, a web-service to analyze proteomics and metabolomics data with technical and biological replicates. The software takes advantage of available replicate variance data to derive more robust and informative differential expression and abundance statistics, whisker plot and PCA visualizations for omics data interpretation. All results, including interactive ranking tables, 2D and 3D PCA visualizations, bar charts and heat maps are generated automatically within few minutes for a typical dataset.

## 2 Workflow and methods

Analyzing omics data with RepExplore requires only the upload of a tab-delimited dataset containing both technical and biological replicates (all parameter settings on the web-interface are optional). The data is processed automatically and the results can be explored interactively in the web-browser.

*Input*: The only input required for RepExplore is a pre-processed proteomics or metabolomics dataset of log-scale intensity measurements in tab-delimited format with labels for biological and technical replicates (example data can be downloaded or analyzed directly on the main web-interface). Optionally, the user can choose to include further normalization procedures, e.g. to ensure that all samples have the same median value (using a median scaling normalization) or to remove dependencies between the signal variance and average signal intensity (using a variance-stabilizing normalization, see Huber *et al.*, 2002).

*Processing*: After submitting an analysis task, the data is processed in the background and a temporary status page is loaded, redirecting the user to the results page after a short waiting time (typically up to a few minutes depending on the dataset size; for large datasets with a limit of 100 MB the status page can be bookmarked). During the statistical data processing, information on measurement uncertainty derived from the variance across technical replicates is exploited using the probability of positive log ratio (PPLR) statistic (Liu *et al.*, 2006; Pearson *et al.*, 2009) to score the differential abundance/expression of biomolecules across the biological conditions. This method takes both summarized point estimates and variation across the replicates into account to obtain a more robust ranking of biomolecules [for comparison, results on the mean-summarized replicates are generated additionally by applying the widely used empirical Bayes moderated t-statistic, here referred to as *eBayes* (Smyth, 2004)]. Similarly, to generate PCA results, the replicate variance data is extracted and used to reduce the influence of noise on the PCA computation (see Sanguinetti *et al.*, 2005).

*Output*: The main result of a submitted analysis is an interactive, sortable ranking table, listing the PPLR and eBayes significance scores and the fold-changes as effect size measure for each biomolecule and allowing the user to generate whisker plots for all table entries of interest. If the user has chosen to generate a PCA visualization, a 2D plot of the first two principal components is shown, revealing potential grouping patterns among the samples or facilitating the recognition of outlier samples. Additionally, the user can view a navigable 3D PCA visualization (Glaab *et al.*, 2010) of the first three principal components by using a VRML browser plugin or an offline VRML-viewer (see Tutorial section on the web-page). Finally, to investigate the separability of sample sub-groups a web-based, interactive heat map visualization using average linkage hierarchical clustering is provided for the top-ranked biomolecules (Deu-Pons *et al.*, 2014).

*Methods and previous validation*: In functional genomics datasets the measured signal for a biomolecule on logarithmic scale is commonly assumed to have an approximate normal distribution (Karpievitch *et al.*, 2009; Sabatine *et al.*, 2005; Sjögren *et al.*, 2007) and to depend on the mean expression/abundance level *μ_i_* and the between-replicate variance *λ_i_* for biological conditions indexed by *i*. If the technical replicate variance *ν_ij_* for condition *i* and replicate *j* is taken into account additionally and assumed to follow a normal distribution centered at zero, the measured signal *y_ij_* can be modeled as follows (Liu *et al.*, 2006):

(1) $$y_{ij}∼N(\mu_{i},\lambda_{i}+\nu_{ij})$$

where the parameters *μ_i_* and *λ_i_* are to be determined. The PPLR approach estimates these parameters using a variational Expectation-Maximization (EM) algorithm, modeling them as independent and *λ* as shared across the biological conditions. The parameter estimates are then used to calculate a differential expression/abundance score, reflecting the PPLR between specified conditions in the input data.

In the same spirit, to reduce the influence of technical noise in PCA, a further dedicated approach has been developed to exploit replicate variance information for PCA computation (Sanguinetti *et al.*, 2005). This method is derived from the interpretation of PCA as the maximum likelihood solution of a probabilistic factor analysis model (Tipping and Bishop, 1999) into which the technical variance is integrated as an additional term (for the detailed derivation, see Sanguinetti *et al.*, 2005). Optimal model parameters are again estimated using an iterative EM algorithm.

These statistical methods have previously been validated on benchmark omics datasets, resulting in improved accuracy in identifying differential abundance patterns (Liu *et al.*, 2006) and tighter sample clusterings (Sanguinetti *et al.*, 2005). In the Supplementary Material, we use multiple proteomic and metabolomic datasets to compare the results obtained from the PPLR method with the eBayes approach, a modification of the classical t-statistic using an empirical Bayes method to shrink the estimated sample variances towards a pooled estimate, providing a more stable inference for small numbers of samples (Smyth, 2004). As a final supplemental analysis, we compare the PPLR results obtained for different numbers of technical replicates on simulated data, showing that the ranking statistics improve with increasing numbers of replicates.

## 3 Results

To illustrate RepExplore’s features and the results obtainable on typical experimental data, we have applied the software to a metabolomics dataset comparing wild-type samples from the plant *Arabidopsis thaliana* against the mutant *mapk phosphatase 1* (*mkp1*), which is more resistant to bacterial infection (Anderson *et al.*, 2014, see datasets overview in the Supplementary Material).

**Fig. 1.**
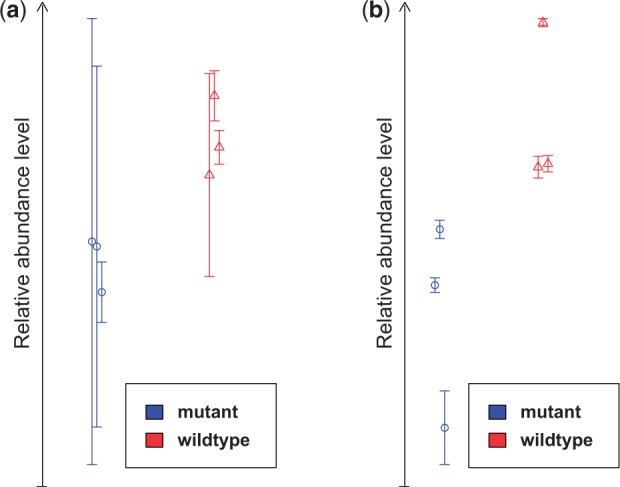
**(a**) Whisker plot for the top differentially abundant metabolite (l-valine) in the Arabidopsis dataset according to the eBayes approach applied to the mean-summarized replicates; (**b**) Whisker plot for the top differentially abundant metabolite (l-proline) according to the PPLR score (circle and triangle symbols represent the sample averages of mutant, resp. wild-type samples, vertical lines represent the technical error per biological sample)

As shown in the whisker plot in Figure 1a, for the top-ranked metabolite identified using a standard eBayes analysis with mean-summarized intensities (l-valine) the overlap of the value ranges for the technical replicates across the two sample groups covers the complete value range of the wild-type samples (only the summarized intensity values are non-overlapping and would suggest a significant difference in the metabolite abundance between the groups). By contrast, for the top-ranked metabolite according to the PPLR score (l-proline), the value ranges of the technical replicates do not display any overlap across the sample groups and the overall replicate variance is significantly smaller (see Fig. 1b). Thus, the whisker plots reveal that the evidence for the induction of l-proline is more reliable than for l-valine, highlighting the benefit of accounting for replicate variance information within the differential abundance statistic.

Ranking tables of metabolites comparing the PPLR and eBayes statistics, heat map visualizations of the metabolite abundance differences between the knockdown and wild-type samples, and further whisker plots for this and other datasets are provided in the Supplementary Materials. The same metabolomics and proteomics example datasets can also be analyzed in an automated fashion on the RepExplore web-application, which enables an interactive exploration of the results (ranking tables are sortable and support the generation of whisker plots for chosen metabolites; the 3D PCA plots provides zoom, pan and rotate functionality, and meta-information is displayed when clicking on a chosen column/row entry in a heat map or on a data point in the 3D plots).

In summary, RepExplore interlinks the automated application of statistical analyses exploiting technical replicate variance information with web-based features to facilitate data exploration via interactive ranking tables and visualizations of the differential expression/abundance patterns. In addition to the public web-application, an exposed programmatic web-service API can be used to control the software, enabling an efficient analysis of multiple large-scale omics datasets.

## 4 Implementation

Statistical data processing and analysis methods were all implemented in the R statistical programming language. The web-application providing access to these statistics is written in PHP and runs on an Apache web-server. To guide the user on how to use the software, a detailed tutorial, help windows for specific features and example datasets from different case/control and wild-type/knockout studies are provided on the web page at http://www.repexplore.tk.

*Conflict of Interest*: none declared.

## Supplementary Material

Supplementary Data
