# Supplementary material for: RepExplore: addressing technical replicate variance in proteomics and metabolomics data analysis
Source: Bioinformatics. 2015 Feb 28;31(13):2235–7. doi: 10.1093/bioinformatics/btv127 (PMC4481852; doi:10.1093/bioinformatics/btv127)
Supplement: Supplementary Data [file supp_31_13_2235__index.html]

RepExplore: Addressing technical replicate variance in proteomics and metabolomics data analysis — RepExplore: addressing technical replicate variance in proteomics and metabolomics data analysis — RepExplore: addressing technical replicate variance in proteomics and metabolomics data analysis — Supplementary Data 

# RepExplore: addressing technical replicate variance in proteomics and metabolomics data analysis

## Supplementary Data

files

**Files in this Data Supplement:**

- Supplementary Data - pdf file
