# Supplementary material for: RepExplore: addressing technical replicate variance in proteomics and metabolomics data analysis
Source: Bioinformatics. 2015 Feb 28;31(13):2235–7. doi: 10.1093/bioinformatics/btv127 (PMC4481852; doi:10.1093/bioinformatics/btv127)
Supplement: Supplementary Data [file supp_btv127_repexplore_supplmat_revision.pdf]

RepExplore: Addressing technical replicate variance in proteomics and metabolomics data analysis — Supplementary Material

Enrico Glaab   Reinhard Schneider

Contents

1 Datasets and pre-processing **2**

2 Analysis results on test datasets **3**

2.1 Results for the Arabidopsis dataset by Anderson et al. 3
2.2 Results for the Arabidopsis dataset by Böttcher et al. 5
2.3 Results for the Alzheimer's dataset by Nagele et al. 7
2.4 Results for the Parkinson's dataset by Han et al. 9
2.5 Results for the Diabetes mellitus type 1 dataset by Koo et al. 11
2.6 Results for the Breast cancer dataset by Nagele et al. 13

3 Simulation experiment for increasing numbers of technical replicates **15**

1 Datasets and pre-processing

To illustrate the functionality of the RepExplore software, the web-application provides example analyses with 6 public omics datasets derived from comparative wild-type vs. knockout/mutant studies and human disease-related case/control studies. An overview of these datasets, including the data type, sample numbers for the biological and technical replicates, and references, is provided in Table S1. The datasets represent different levels of complexity, quality and structuredness in order to exemplify the range of outputs obtained with RepExplore on diverse types of data.

Before applying the statistical analyses to obtain the ranking tables and visualizations provided in the following sections, the log-scaled intensity measurements from all datasets underwent a median-scaling normalization to ensure that all samples have the same median value. For the protoeomics data derived from the ProtoArray platform, prior to the median-scaling the data from different batches was additionally normalized and adjusted using the Cyclic Loess and ComBat approaches [Ballman *et al.*, 2004, Johnson *et al.*, 2007]. For the metabolomics data from the studies on *Arabidopsis thaliana*, except for the additional median-scaling the data pre-processing from the original study was retained [Böttcher *et al.*, 2009, Anderson *et al.*, 2014].

Dataset	Type	# samples $\times$ # technical replicates	References
Arabidopsis thaliana	Metabolomics	3×2 (mutant) / 3×2 (wild-type)	[Anderson *et al.*, 2014]
Arabidopsis thaliana	Metabolomics	4×2 (knockout) / 4×2 (wild-type)	[Böttcher *et al.*, 2009]
Alzheimer's disease	Proteomics	50×2 (case) / 40×2 (control)	[Nagele *et al.*, 2011]
Parkinson's disease	Proteomics	29×2 (case) / 40×2 (control)	[Han *et al.*, 2012]
Type 1 Diabetes mellitus	Proteomics	16×2 (case) / 27×2 (control)	[Koo *et al.*, 2014]
Breast cancer	Proteomics	30×2 (case) / 40×2 (control)	[Nagele *et al.*, 2011]

Table S1: **Overview of used omics datasets and sample sizes**

2 Analysis results on test datasets

In the following, the differential abundance ranking tables, heat maps and example whisker plots are provided for the test datasets presented in the “Datasets and pre-processing” section. Interactive, web-based versions of the heat maps and 3D PCA plots for the same datasets can be generated in an automated fashion on the RepExplore web-interface at `http://lcsb-portal.uni.lu/repexplore`.

2.1 Results for the Arabidopsis dataset by Anderson et al.

Identifier	logFC	PPLR	P-like value	eBayes T-score	eBayes P-value	eBayes Q-value
L-proline	-1.25504	1	0	-3.32209	0.011864	0.198881
D-Lyxopyranose	-0.3941	0.999999	1.18E-06	-2.88412	0.022307	0.198881
2-ketogluconic acid	0.424322	5.32E-06	5.32E-06	2.522033	0.038164	0.254201
L-threonine	-0.4454	0.999979	2.06E-05	-2.81044	0.02486	0.198881
sucrose	-0.95815	0.999884	0.000116	-1.79064	0.114447	0.452684
glycine	-0.37293	0.999797	0.000203	-1.74426	0.122602	0.452684
L-serine	-0.44678	0.999673	0.000327	-2.94155	0.020509	0.198881
2,4-Dihydroxybutanoic acid	0.32488	0.001134	0.001134	1.813018	0.110699	0.452684
L-Pyroglutamic acid	-0.26869	0.99854	0.00146	-2.00508	0.083046	0.39862
D-galactose	-0.2652	0.996406	0.003594	-1.69164	0.132518	0.454348
myo-inositol	0.208944	0.024355	0.024355	1.225992	0.258034	0.568664
urea	-0.59337	0.942231	0.057769	-1.30197	0.23225	0.568664
xylitol	-0.20737	0.9044	0.0956	-1.56415	0.159735	0.479205
beta-alanine	-0.31778	0.899886	0.100114	-2.43291	0.043619	0.254201
L-Lactic acid	0.726053	0.109865	0.109865	2.373908	0.047663	0.254201
glycolic acid	0.276557	0.113517	0.113517	1.236622	0.254287	0.568664
3-Glycerophosphate	0.512701	0.113806	0.113806	1.607713	0.149901	0.479205
fumaric acid	-0.13642	0.870409	0.129591	-0.84713	0.423651	0.701216
Lilioside B	-0.18224	0.859448	0.140552	-1.18409	0.273258	0.568664
o-Toluic acid	0.276948	0.141876	0.141876	1.154819	0.284332	0.568664

Table S2: **Differential abundance ranking for the Arabidopsis dataset by Anderson et al.** For each of the top 20 metabolites, the logarithmic fold-change between wild-type and knockout samples (logFC), the probability of positive likelihood ratio (PPLR) statistic, the transformation of the PPLR into a P-like significance score (min(PPLR,1-PPLR)) and the empirical Bayes moderated t-test (eBayes) p-value and adjusted p-values (Q-value) using the Benjamini-Hochberg method [Benjamini & Hochberg, 1995] are reported.

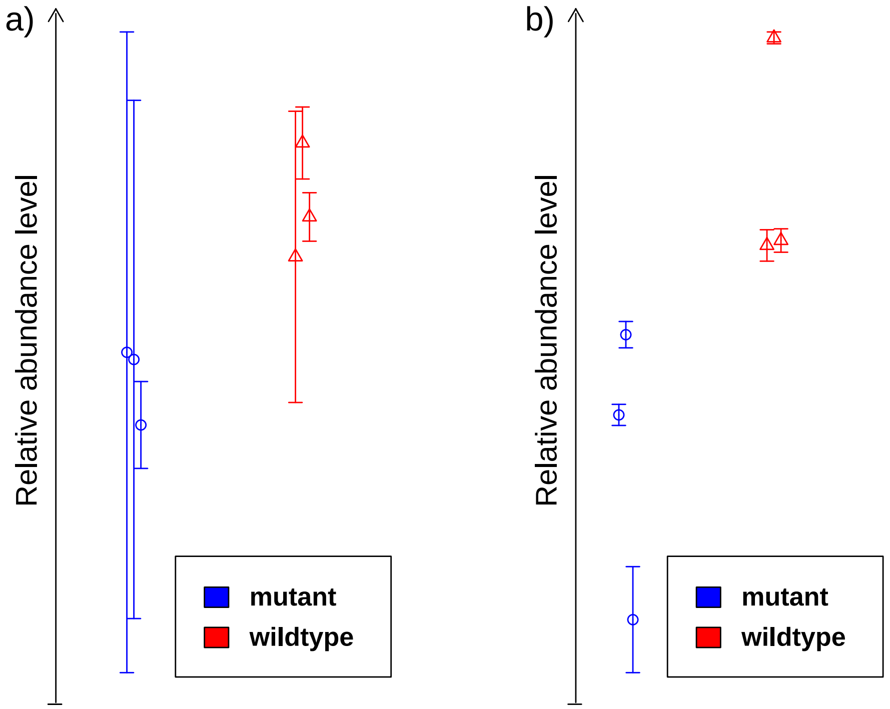

Figure S1: a) Whisker plot for the top differentially abundant metabolite (L-valine) in the Arabidopsis dataset by Anderson et al. according to the eBayes approach applied to the mean-summarized replicates; b) Whisker plot for the top differentially abundant metabolite (L-proline) in the Arabidopsis dataset according to the PPLR score (circle and triangle symbols represent the sample averages of mutant, resp. wild-type samples, see also the description in the main manuscript).

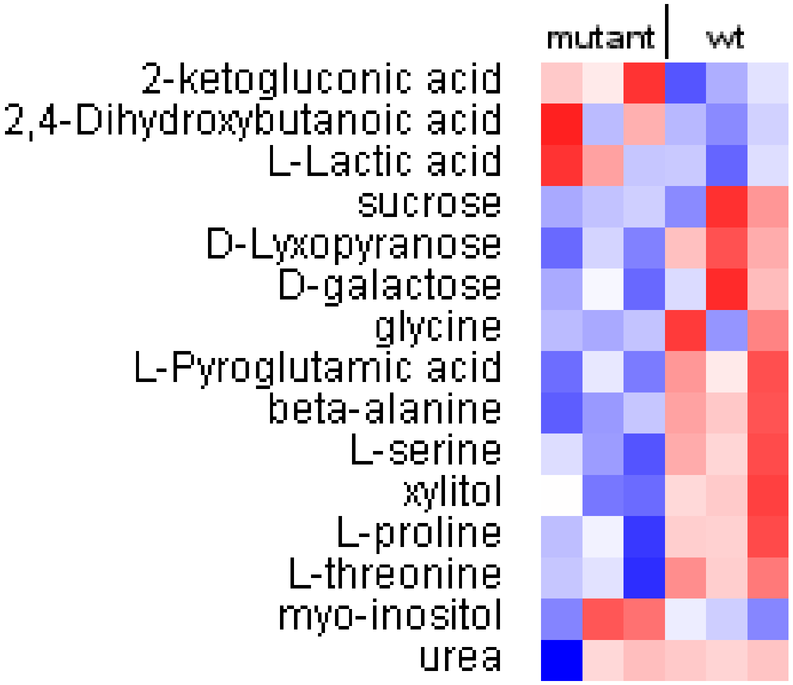

Figure S2: Heat map visualization of the top 15 metabolite abundance differences between mutant and wild-type (wt) samples from the Arabidopsis dataset by Anderson et al. according to the PPLR score as an example output of RepExplore, using average linkage hierarchical clustering for both rows and columns (rows = metabolites, columns = samples). Since error bars cannot be included in the heat map, the colors provide a representation of the row Z-scores of the intensities averaged across the technical replicates (blue = low relative abundance, red = high relative abundance).

2.2 Results for the Arabidopsis dataset by Böttcher et al.

Identifier	logFC	PPLR	P-like value	eBayes T-score	eBayes P-value	eBayes Q-value
6-Hexosyloxyindole-3-carboxylic acid	-0.91487	0.951322	0.048678	-8.82712	7.07E-07	2.40E-06
gammaGlu-Cys(IAN)	1.505109	0.068014	0.068014	12.76627	9.08E-09	5.14E-08
2-Formamidophenyl-2'-thiazolylketone	1.421462	0.068909	0.068909	16.43669	3.98E-10	6.76E-09
Methoxy-Dihydroascorbigen Hexoside	-0.88891	0.924531	0.075469	-12.3733	1.33E-08	5.64E-08
Methyl indole-3-carboxylate	1.34855	0.100267	0.100267	14.63591	1.69E-09	1.44E-08
Hexosyl indole-3-carboxylate	0.470099	0.144167	0.144167	6.564687	1.74E-05	3.71E-05
Camalexin	0.594932	0.146795	0.146795	8.403123	1.23E-06	2.99E-06
Indol-3-ylmethylamine	-0.37881	0.850914	0.149086	-5.42974	0.000112	0.00019
Hydroxy-Dihydroascorbigen Hexoside	-0.3078	0.843037	0.156963	-4.11137	0.001208	0.001579
3-Hydroxy-3-(thiazol-2-yl)indolin-2-one	0.677226	0.182179	0.182179	8.443786	1.17E-06	2.99E-06
1-Methoxy-indol-3-ylmethylglucosinolate	-0.55825	0.734481	0.265519	-5.48237	0.000102	0.00019
gammaGlu-Cys(4MeOI3M)-Gly	-0.52319	0.710045	0.289955	-4.68746	0.000416	0.000643
Ascorbigen	0.473502	0.302809	0.302809	4.239087	0.000951	0.001347
Indol-3-ylmethylglucosinolate	-0.16508	0.614303	0.385697	-1.98107	0.068973	0.078169
gammaGlu-Cys(IAN)-Glu	0.176553	0.416749	0.416749	2.274575	0.04038	0.049033
Dihydroascorbigen Hexoside	-0.16167	0.569063	0.430937	-1.30045	0.215857	0.229348
4-Methoxy-indol-3-ylmethylglucosinolate	-0.05377	0.54421	0.45579	-0.68201	0.507112	0.507112

Table S3: **Differential abundance ranking for the Arabidopsis dataset by Böttcher et al.** For each metabolite, the logarithmic fold-change between wild-type and knockout samples (logFC), the probability of positive likelihood ratio (PPLR) statistic, the transformation of the PPLR into a P-like significance score (min(PPLR,1-PPLR)) and the empirical Bayes moderated t-test (eBayes) p-value and adjusted p-values (Q-value) using the Benjamini-Hochberg method [Benjamini & Hochberg, 1995] are reported.

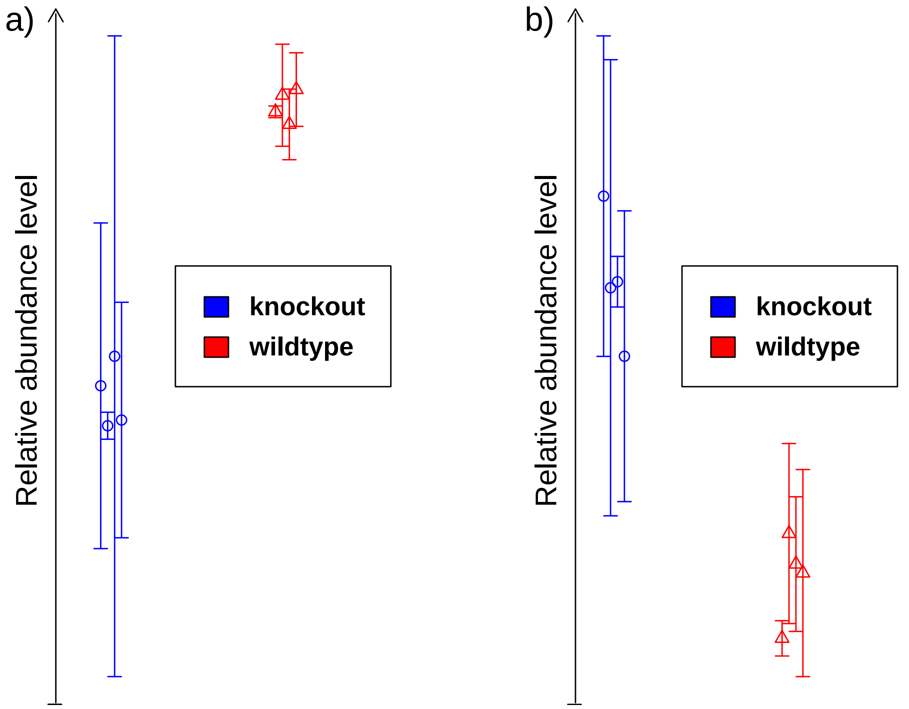

Figure S3: a) Whisker plot for the top differentially abundant metabolite (2-Formamidophenyl-2'-thiazolylketone) in the Arabidopsis dataset by Böttcher et al. according to the eBayes approach applied to the mean-summarized replicates; b) Whisker plot for the top differentially abundant metabolite (6-Hexosyloxyindole-3-carboxylic acid) in the Arabidopsis dataset according to the PPLR score.

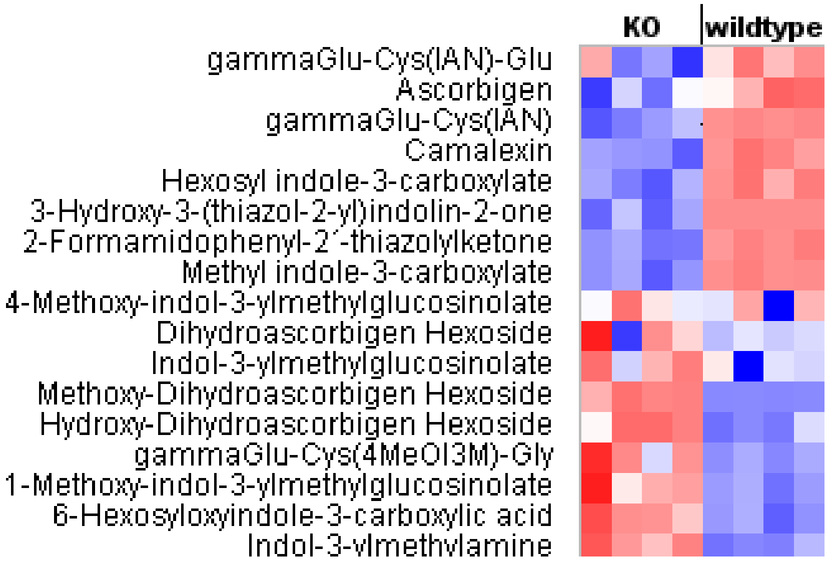

Figure S4: Heat map visualization of metabolite abundance differences between knockdown (KO) and wild-type samples from the Arabidopsis dataset by Böttcher et al. according to the PPLR score as an example output of RepExplore, using average linkage hierarchical clustering for both rows and columns (rows = metabolites, columns = samples). Since error bars cannot be included in the heat map, the colors provide a representation of the row Z-scores of the intensities averaged across the technical replicates (blue = low relative abundances, red = high relative abundances).

2.3 Results for the Alzheimer's dataset by Nagele et al.

Identifier	logFC	PPLR	P-like value	eBayes T-score	eBayes P-value	eBayes Q-value
ZCCHC11	-0.82188	0.934938	0.065062	-2.11936	0.036753	0.999817
IGL@	0.164649	0.074264	0.074264	2.517434	0.013551	0.999817
PPP1R8	-0.8573	0.925412	0.074588	-2.23458	0.027866	0.999817
PTCD2	0.388974	0.085309	0.085309	6.284214	1.08E-08	9.55E-05
WDR5 1	0.388674	0.093464	0.093464	2.554316	0.012283	0.999817
UBE2S	0.455524	0.125551	0.125551	2.563273	0.011991	0.999817
HSPC111	0.427424	0.140155	0.140155	2.742388	0.00733	0.999817
IGLV1-44	0.154999	0.143192	0.143192	2.457183	0.015878	0.999817
ECH1	-0.65673	0.852911	0.147089	-2.27502	0.025227	0.999817
RPL23A	0.370149	0.149565	0.149565	2.514571	0.013654	0.999817
VRK1	0.431174	0.150777	0.150777	2.5562	0.012221	0.999817
ASAH1	-0.6047	0.8419	0.1581	-2.37784	0.019484	0.999817
IGLC1	0.166299	0.158138	0.158138	2.507807	0.013901	0.999817
FRMD8	0.395649	0.159695	0.159695	4.159935	7.15E-05	0.21003
PIN4	0.324074	0.160556	0.160556	2.268685	0.025626	0.999817
NEK7	0.331874	0.171496	0.171496	2.596738	0.010957	0.999817
CPB1	-0.66148	0.828356	0.171644	-2.49034	0.014557	0.999817
PADI4	0.390599	0.173868	0.173868	2.790781	0.006393	0.999817
ASXL1	0.416499	0.178777	0.178777	2.578754	0.011502	0.999817
CSNK2A2	0.401774	0.183065	0.183065	2.472716	0.015246	0.999817

Table S4: **Differential abundance ranking for the Alzheimer's dataset by Nagele et al.** For each of the top 20 proteins, the logarithmic fold-change between Alzheimer's disease and non-demented control samples (logFC), the probability of positive likelihood ratio (PPLR) statistic, the transformation of the PPLR into a P-like significance score (min(PPLR,1-PPLR)) and the empirical Bayes moderated t-test (eBayes) p-value and adjusted p-values (Q-value) using the Benjamini-Hochberg method [Benjamini & Hochberg, 1995] are reported.

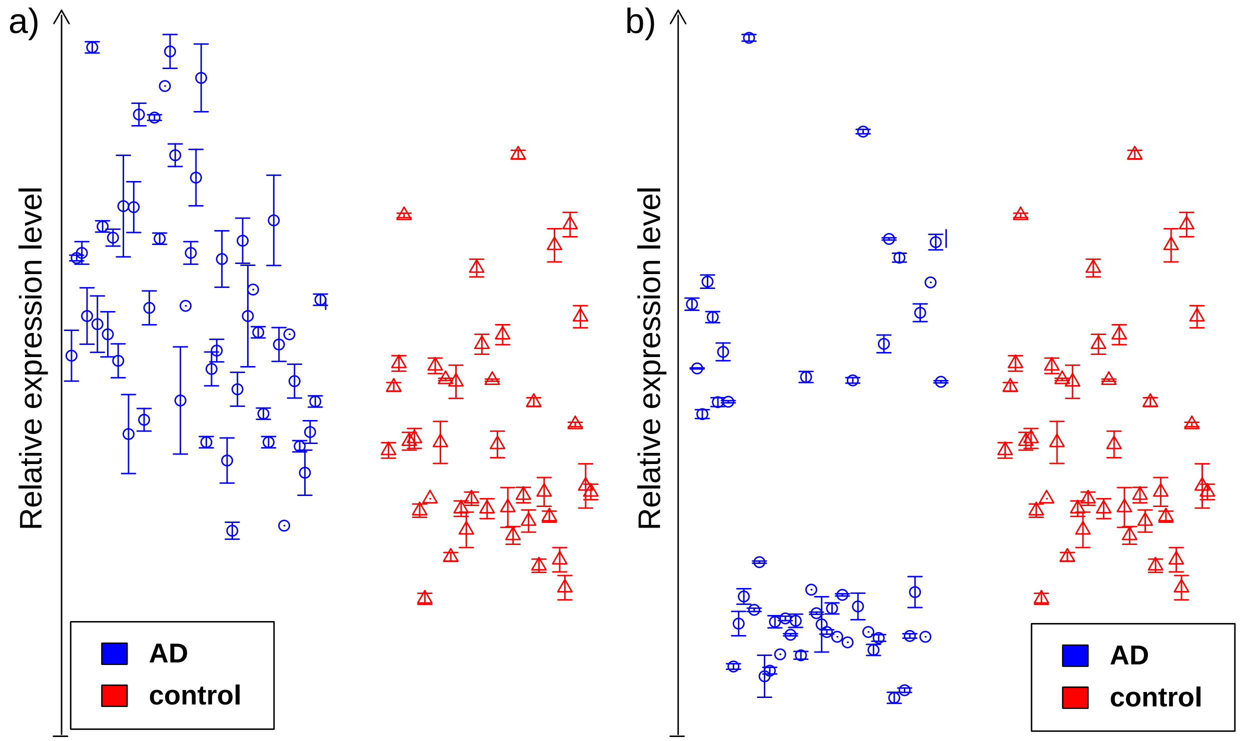

Figure S5: a) Whisker plot for the top differentially expressed protein (pentatricopeptide repeat domain 2, PTCD2) in the Alzheimer's dataset by Nagele et al. according to the eBayes approach applied to the mean-summarized replicates; b) Whisker plot for the top differentially expressed protein (zinc finger, CCHC domain containing 11, ZCCHC11) in the Alzheimer's dataset according to the PPLR score (interestingly, the plot suggests a potential two group clustering pattern among the Alzheimer's disease samples).

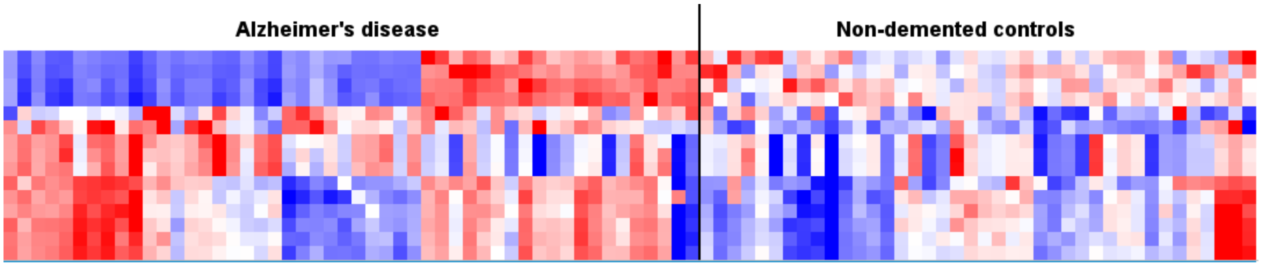

Figure S6: Heat map visualization of the top 15 protein abundance differences between Alzheimer's disease and non-demented control samples from the study by Nagele et al. according to the PPLR score as an example output of RepExplore, using average linkage hierarchical clustering for both rows and columns (rows = proteins, columns = samples; in order to view the protein identifiers, please use the interactive version of the heat map on the RepExplore web-application). Since error bars cannot be included in the heat map, the colors provide a representation of the row Z-scores of the intensities averaged across the technical replicates (blue = low relative abundance, red = high relative abundance).

2.4 Results for the Parkinson's dataset by Han et al.

Identifier	logFC	PPLR	P-like value	eBayes T-score	eBayes P-value	eBayes Q-value
Ig gamma-1 chain C region,1	-0.07405	0.852518	0.147482	-1.05034	0.297104	0.999988
IGL@	-0.07232	0.838619	0.161381	-0.97107	0.334788	0.999988
IGLV1-44	-0.07047	0.718128	0.281872	-0.98294	0.328954	0.999988
SULF1	-0.27208	0.69864	0.30136	-0.72819	0.46888	0.999988
FRMD8	-0.1688	0.668733	0.331267	-1.26607	0.209602	0.999988
C9orf86	0.403125	0.334877	0.334877	1.209264	0.230548	0.999988
ZXDC	0.137814	0.336548	0.336548	0.772089	0.442609	0.999988
PPP1R8	0.389017	0.344286	0.344286	1.670579	0.099181	0.999988
Ig gamma-1 chain C region,2	-0.07381	0.638597	0.361403	-1.05034	0.297104	0.999988
MTMR2	0.383737	0.362389	0.362389	1.560011	0.123177	0.999988
SH3GL2	-0.16527	0.631274	0.368726	-0.61811	0.53847	0.999988
PTCD2	-0.13847	0.627931	0.372069	-1.67611	0.098087	0.999988
LRRC42	0.318176	0.375792	0.375792	2.057739	0.043265	0.999988
BC015833	-0.06348	0.615404	0.384596	-0.90942	0.366187	0.999988
STX8	-0.10392	0.614509	0.385491	-0.74011	0.461659	0.999988
FAM136A	-0.0902	0.61224	0.38776	-0.36207	0.718369	0.999988
Centromere protein R	-0.10028	0.610877	0.389123	-0.3977	0.692039	0.999988
FN1	-0.09617	0.606569	0.393431	-0.91227	0.364698	0.999988
RAB10	-0.08013	0.59929	0.40071	-0.79387	0.429901	0.999988
FCGR3A	-0.46116	0.597481	0.402519	-1.18981	0.23806	0.999988

Table S5: **Differential abundance ranking for the Parkinson's dataset by Han et al.** For each of the top 20 proteins, the logarithmic fold-change between Parkinson's disease and non-demented control samples (logFC), the probability of positive likelihood ratio (PPLR) statistic, the transformation of the PPLR into a P-like significance score (min(PPLR,1-PPLR)) and the empirical Bayes moderated t-test (eBayes) p-value and adjusted p-values (Q-value) using the Benjamini-Hochberg method [Benjamini & Hochberg, 1995] are reported.

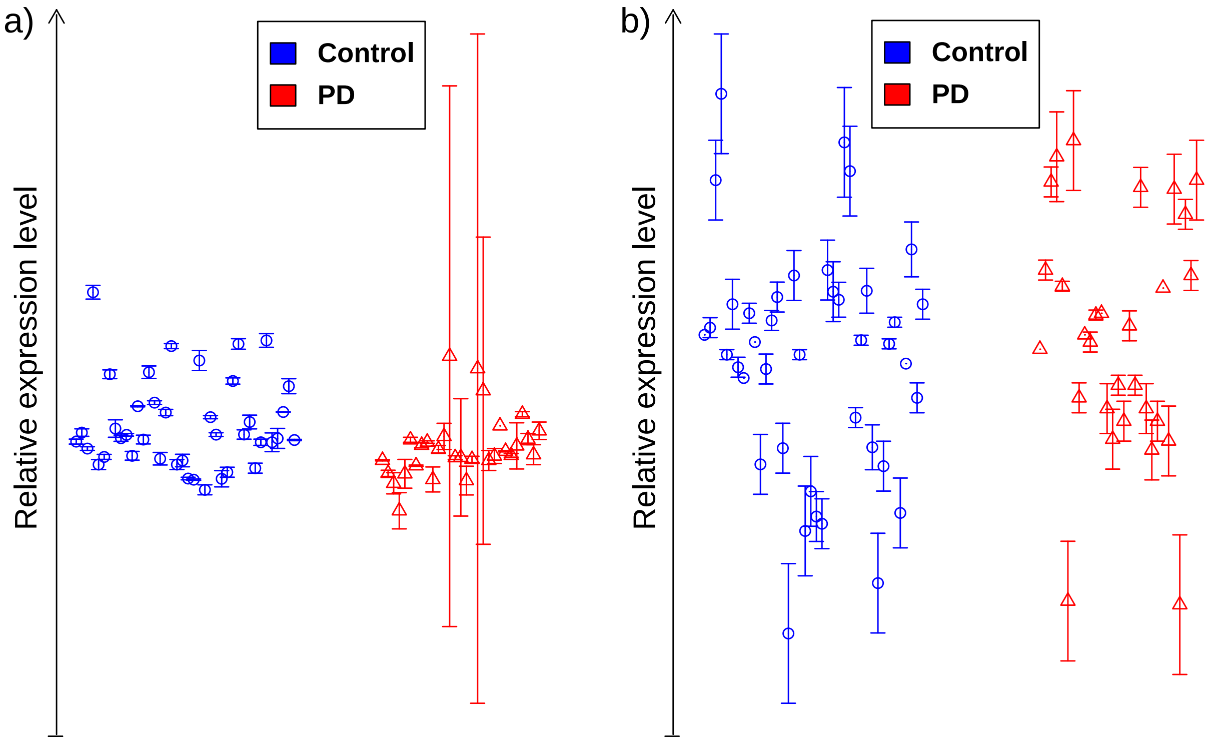

Figure S7: a) Whisker plot for the top differentially expressed protein (spermatogenesis associated 1, SPATA1) in the Parkinson's dataset by Han et al. according to the eBayes approach applied to the mean-summarized replicates; b) Whisker plot for the top differentially expressed protein (Ig gamma-1 chain C region,1) in the Parkinson's dataset according to the PPLR score.

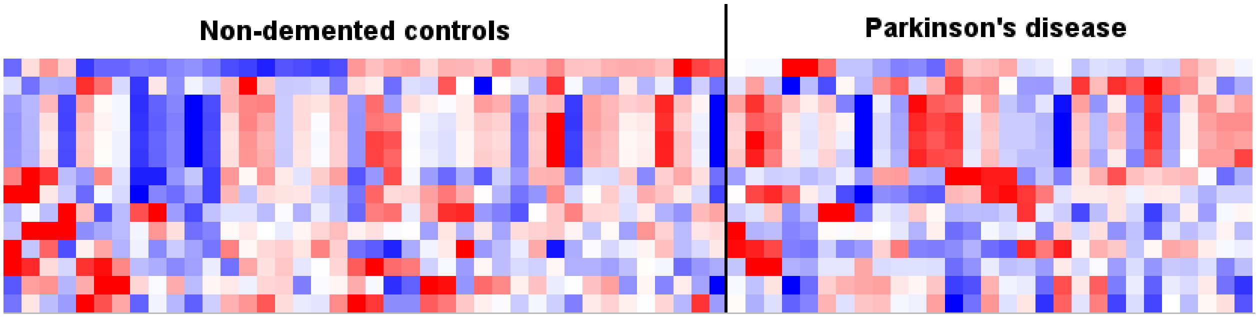

Figure S8: Heat map visualization of the top 15 protein abundance differences between Parkinson's disease and non-demented control samples from the study by Han et al. according to the PPLR score as an example output of RepExplore, using average linkage hierarchical clustering for both rows and columns (rows = proteins, columns = samples; in order to view the protein identifiers, please use the interactive version of the heat map on the RepExplore web-application). Since error bars cannot be included in the heat map, the colors provide a representation of the row Z-scores of the intensities averaged across the technical replicates (blue = low relative abundance, red = high relative abundance). No clear discriminative pattern could be identified in this dataset.

2.5 Results for the Diabetes mellitus type 1 dataset by Koo et al.

Identifier	logFC	PPLR	P-like value	eBayes T-score	eBayes P-value	eBayes Q-value
Glutamate decarboxylase 2	-0.68739	0.999012	0.000988	-2.97093	0.004878	0.999863
RBPJ	0.443119	0.016127	0.016127	1.042389	0.30315	0.999863
ADSSL1	0.382702	0.029873	0.029873	1.705104	0.095506	0.999863
FAM126B	0.352418	0.067281	0.067281	1.264616	0.212929	0.999863
TMPRSS4	0.36243	0.076101	0.076101	1.174205	0.246878	0.999863
CENTG2	-0.31243	0.921607	0.078393	-1.33272	0.189755	0.999863
FAM84A	0.542998	0.096786	0.096786	1.492546	0.142974	0.999863
LRRC6	0.536025	0.104446	0.104446	1.86782	0.068722	0.999863
UPK3A	0.248964	0.110485	0.110485	0.843987	0.403422	0.999863
MAP4	-0.19161	0.870581	0.129419	-0.68184	0.499052	0.999863
Glutamate decarboxylase 1	-0.30604	0.866325	0.133675	-1.30953	0.197418	0.999863
KIAA0515	0.308709	0.134459	0.134459	1.138707	0.261231	0.999863
Transcription cofactor HES-6	-0.22568	0.864701	0.135299	-1.43503	0.158628	0.999863
SURF5	-0.20821	0.856453	0.143547	-0.63267	0.530353	0.999863
GOLGA4	-0.24525	0.856199	0.143801	-0.84129	0.404915	0.999863
RPS21	-0.28152	0.847693	0.152307	-1.22558	0.227134	0.999863
HBZ	-0.16466	0.845446	0.154554	-0.77599	0.442071	0.999863
MESDC2	0.26944	0.155257	0.155257	1.332158	0.189938	0.999863
AMOTL2	0.321688	0.162718	0.162718	1.069946	0.290708	0.999863
LOC133874	0.281514	0.162964	0.162964	0.955189	0.344907	0.999863

Table S6: **Differential abundance ranking for the Diabetes mellitus type 1 dataset by Koo et al.** For each of the top 20 proteins, the logarithmic fold-change between Diabetes mellitus type 1 and control samples (logFC), the probability of positive likelihood ratio (PPLR) statistic, the transformation of the PPLR into a P-like significance score (min(PPLR,1-PPLR)) and the empirical Bayes moderated t-test (eBayes) p-value and adjusted p-values (Q-value) using the Benjamini-Hochberg method [Benjamini & Hochberg, 1995] are reported.

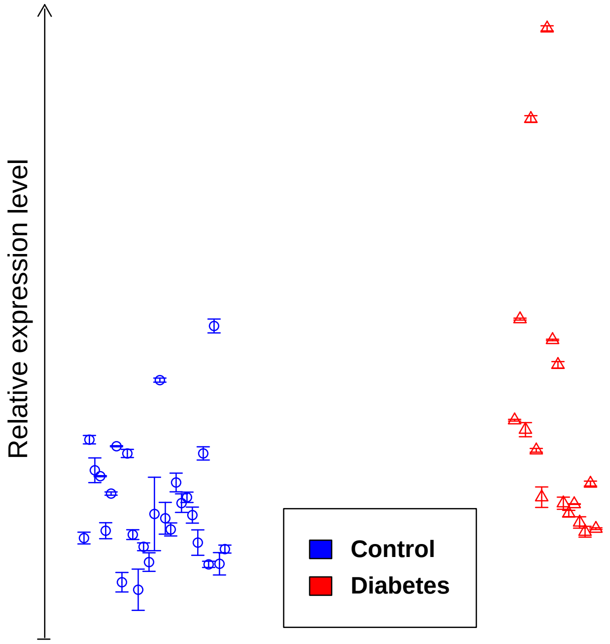

Figure S9: Whisker plot for the top differentially expressed protein (Glutamate decarboxylase 2) in the Diabetes mellitus type 1 dataset by Koo et al. according to the PPLR approach and eBayes approach applied to the mean-summarized replicates, i.e. for this dataset the methods agreed on the top-ranked protein.

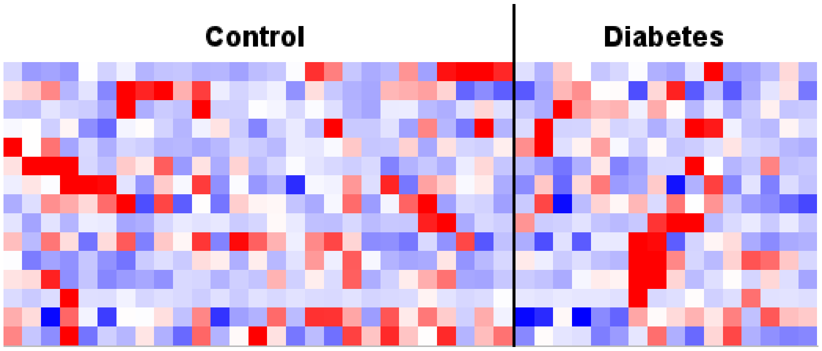

Figure S10: Heat map visualization of the top 15 protein abundance differences between Diabetes mellitus type 1 and control samples from the study by Koo et al. according to the PPLR score as an example output of RepExplore, using average linkage hierarchical clustering for both rows and columns (rows = proteins, columns = samples; in order to view the protein identifiers, please use the interactive version of the heat map on the RepExplore web-application). Since error bars cannot be included in the heat map, the colors provide a representation of the row Z-scores of the intensities averaged across the technical replicates (blue = low relative abundance, red = high relative abundance). No clear discriminative pattern could be identified in this dataset.

2.6 Results for the Breast cancer dataset by Nagele et al.

Identifier	logFC	PPLR	P-like value	eBayes T-score	eBayes P-value	eBayes Q-value
RAB10	-0.444	0.941607	0.058393	-5.62507	3.28E-07	0.000481
Ig gamma-1 chain C region	0.053083	0.211446	0.211446	0.715156	0.476811	1
SULF1	0.304	0.234456	0.234456	0.865487	0.389628	1
phenylalanine hydroxylase (PAH)	-0.3805	0.763347	0.236653	-5.99657	7.22E-08	0.000232
UPF0471 protein C1orf63 homolog	-0.35767	0.736722	0.263278	-1.8612	0.066771	1
FAM19A5	-0.41158	0.732895	0.267105	-6.08903	4.93E-08	0.000232
CMAH	-0.45088	0.728873	0.271127	-5.57794	3.96E-07	0.000499
LRRC42	-0.31404	0.720571	0.279429	-2.09005	0.040123	1
UPF0566	-0.22275	0.718586	0.281414	-0.67499	0.501828	1
SPANXE	-0.60383	0.712148	0.287852	-5.34261	1.01E-06	0.000992
SLC7A6OS	-0.48079	0.712057	0.287943	-5.97364	7.93E-08	0.000232
serine dehydratase (SDS)	-0.39633	0.700774	0.299226	-5.6482	2.98E-07	0.000481
LIMCH1	-0.38346	0.697633	0.302367	-1.9904	0.050319	1
TBC1D7	-0.27821	0.680119	0.319881	-1.57685	0.119185	1
C1orf211	-0.45333	0.671183	0.328817	-5.90475	1.05E-07	0.000232
CCNB1IP1	-0.32338	0.667692	0.332308	-1.83314	0.070889	1
PCNA	-0.25571	0.665731	0.334269	-1.21312	0.229022	1
MTMR2	-0.34396	0.649946	0.350054	-1.62947	0.107553	1
PDPK1	-0.31587	0.648061	0.351939	-1.52861	0.130714	1
ERO1LB	-0.262	0.646622	0.353378	-1.41736	0.160662	1

Table S7: **Differential abundance ranking for the Breast cancer dataset by Nagele et al.** For each of the top 20 proteins, the logarithmic fold-change between Breast cancer and control samples (logFC), the probability of positive likelihood ratio (PPLR) statistic, the transformation of the PPLR into a P-like significance score (min(PPLR,1-PPLR)) and the empirical Bayes moderated t-test (eBayes) p-value and adjusted p-values (Q-value) using the Benjamini-Hochberg method [Benjamini & Hochberg, 1995] are reported.

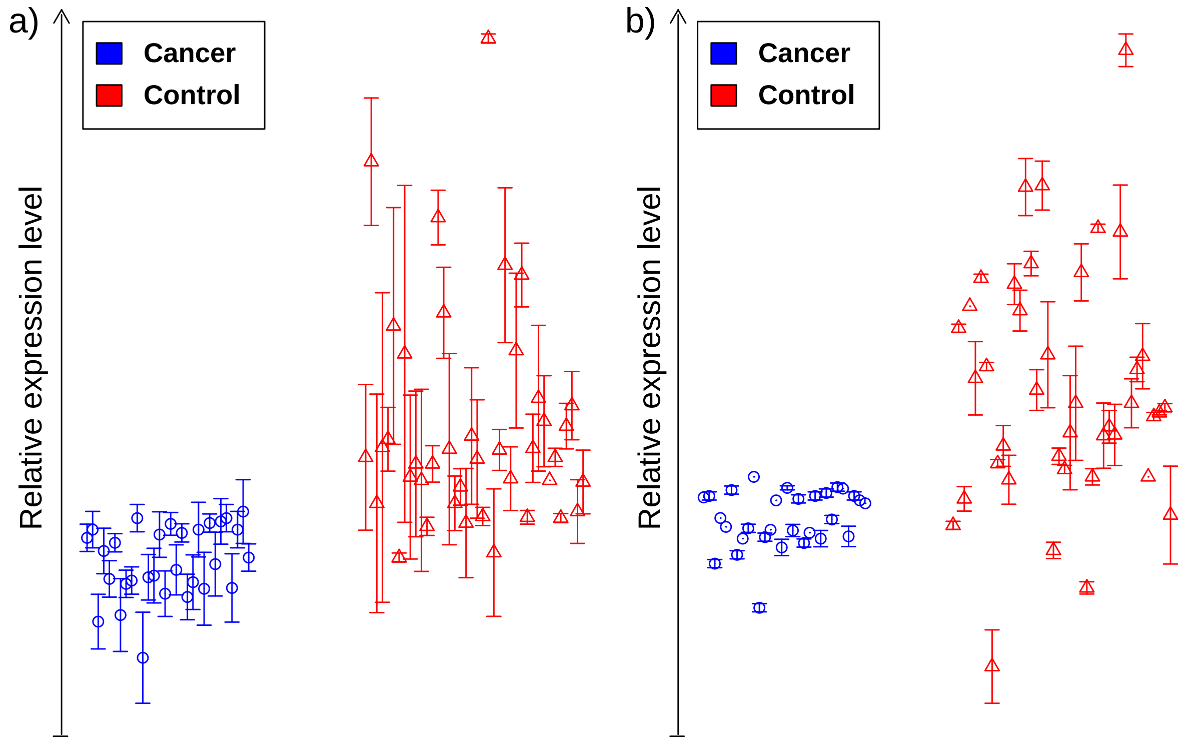

Figure S11: a) Whisker plot for the top differentially expressed protein (FAM19A5) in the Breast cancer dataset by Nagele et al. according to the eBayes approach applied to the mean-summarized replicates; b) Whisker plot for the top differentially expressed protein (RAB10, member RAS oncogene family) in the Breast cancer dataset according to the PPLR score.

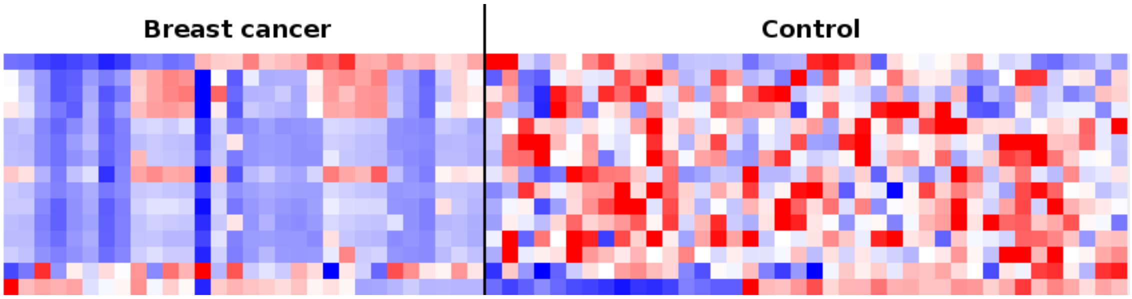

Figure S12: Heat map visualization of the top 15 protein abundance differences between Breast cancer and control samples from the study by Nagele et al. according to the PPLR score as an example output of RepExplore, using average linkage hierarchical clustering for both rows and columns (rows = proteins, columns = samples; in order to view the protein identifiers, please use the interactive version of the heat map on the RepExplore web-application). Since error bars cannot be included in the heat map, the colors provide a representation of the row Z-scores of the intensities averaged across the technical replicates (blue = low relative abundance, red = high relative abundance).

3 Simulation experiment for increasing numbers of technical replicates

Since the added value of the probability of positive likelihood ratio (PPLR) statistic for ranking differentially abundant biomolecules consists in exploiting the measurement variance information from technical replicates, we evaluated whether increasing numbers of technical replicates also result in improved detection of truly altered features by using simulated data with known truly differential attributes. Specifically, we generated a set of simulated normal data with constant standard deviation of $\sigma = 1$, containing 100 samples (here corresponding to the biological replicates) divided into two balanced sample groups (50 samples in group 1 and 50 in group 2) and 1000 features/biomolecules, consisting of 900 uncorrelated, irrelevant biomolecules and 100 known differentially abundant biomolecules (with a fixed effect size of D = 1). Next, simulated technical replicates with additional measurement noise were created for each biological sample by first copying the original simulated data 10 times and then inserting additional random noise into each of the 10 copies (using the function *jitter* in the R Statistical Programming Language). The PPLR statistic was then applied to incrementally expanded, combined subsets of this data corresponding to increasing numbers of technical replicates, from 2 technical measurements per sample up to 10 (adding one further technical replicate per sample to the input dataset in each step). To evaluate the results for the obtained 10 PPLR-rankings of the 1000 features/biomolecules, we quantified the enrichment of the 100 known differentially abundant biomolecules among the top-ranked biomolecules in each of the PPLR-rankings using the normalized Kolmogorov-Smirnov statistic (see [Mootha *et al.*, 2003]; in short, larger values of this statistic correspond to an improved enrichment of the known differential biomolecules among higher-scored entries in the currently considered ranking list). The resulting enrichment statistics for increasing numbers of used technical replicates are shown in the bar chart in Fig. S13 (all enrichment scores were significant with a permutation-based p-value < 0.001). This figure clearly reveals that for the simulated data the enrichment statistic increases strictly monotonously with increasing numbers of replicates, showing that the PPLR approach was able to exploit an added informative value from each series of technical replicates included in the dataset.

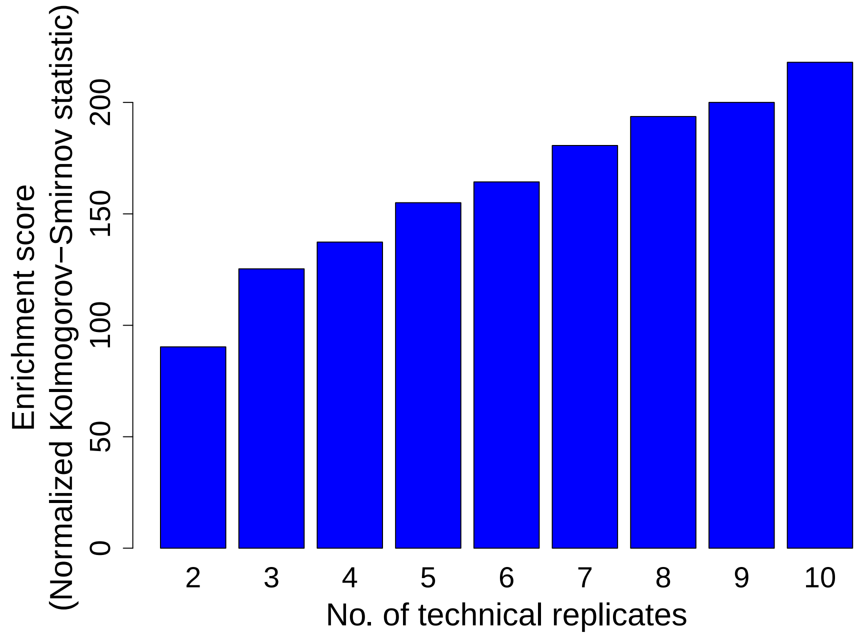

Figure S13: Bar chart showing the enrichment of known differentially abundant biomolecules/features in the simulated data among the PPLR-rankings for increasing numbers of included technical replicates.

References

[Anderson *et al.*, 2014] Anderson, J. C., Wan, Y., Kim, Y.-M., Pasa-Tolic, L., Metz, T. O. & Peck, S. C. (2014) Decreased abundance of type III secretion system-inducing signals in Arabidopsis mkp1 enhances resistance against Pseudomonas syringae. *Proceedings of the National Academy of Sciences,* **111** (18), 6846–6851.

[Ballman *et al.*, 2004] Ballman, K. V., Grill, D. E., Oberg, A. L. & Therneau, T. M. (2004) Faster cyclic loess: normalizing RNA arrays via linear models. *Bioinformatics,* **20** (16), 2778–2786.

[Benjamini & Hochberg, 1995] Benjamini, Y. & Hochberg, Y. (1995) Controlling the false discovery rate: a practical and powerful approach to multiple testing. *Journal of the Royal Statistical Society. Series B (Methodological),* **57**, 289–300.

[Böttcher *et al.*, 2009] Böttcher, C., Westphal, L., Schmotz, C., Prade, E., Scheel, D. & Glawischnig, E. (2009) The multifunctional enzyme CYP71B15 (PHYTOALEXIN DEFICIENT3) converts cysteine-indole-3-acetonitrile to camalexin in the indole-3-acetonitrile metabolic network of Arabidopsis thaliana. *Plant Cell,* **21** (6), 1830–1845.

[Han *et al.*, 2012] Han, M., Nagele, E., DeMarshall, C., Acharya, N. & Nagele, R. (2012) Diagnosis of Parkinson's disease based on disease-specific autoantibody profiles in human sera. *PLoS One,* **7** (2), e32383.

[Johnson *et al.*, 2007] Johnson, W. E., Li, C. & Rabinovic, A. (2007) Adjusting batch effects in microarray expression data using empirical Bayes methods. *Biostatistics,* **8** (1), 118–127.

[Koo *et al.*, 2014] Koo, B. K., Chae, S., Kim, K. M., Kang, M. J., Kim, E. G., Kwak, S. H., Jung, H. S., Cho, Y. M., Choi, S. H., Park, Y. J. *et al.* (2014) Identification of novel autoantibodies in type 1 diabetic patients using a high-density protein microarray. *Diabetes,* **63** (9), 3022–3032.

[Mootha *et al.*, 2003] Mootha, V. K., Lindgren, C. M., Eriksson, K.-F., Subramanian, A., Sihag, S., Lehar, J., Puigserver, P., Carlsson, E., Ridderstråle, M., Laurila, E. *et al.* (2003) Pgc-1α-responsive genes involved in oxidative phosphorylation are coordinately downregulated in human diabetes. *Nature Genetics,* **34** (3), 267–273.

[Nagele *et al.*, 2011] Nagele, E., Han, M., DeMarshall, C., Belinka, B. & Nagele, R. (2011) Diagnosis of Alzheimer's disease based on disease-specific autoantibody profiles in human sera. *PLoS One,* **6** (8), e23112.
